# Video Smoke Detection Method Based on Change-Cumulative Image and Fusion Deep Network

**DOI:** 10.3390/s19235060

**Published:** 2019-11-20

**Authors:** Tong Liu, Jianghua Cheng, Xiangyu Du, Xiaobing Luo, Liang Zhang, Bang Cheng, Yang Wang

**Affiliations:** College of Electronic Science, National University of Defense Technology, Changsha 410073, China; liutong1129@nudt.edu.cn (T.L.); xiangyu_du@163.com (X.D.); luoxiaobing77@126.com (X.L.); zhangliang313417@163.com (L.Z.); chengbang@nudt.edu.cn (B.C.); zhang_missing@163.com (Y.W.)

**Keywords:** video smoke detection, deep learning, object detection, convolutional neural networks

## Abstract

Smoke detection technology based on computer vision is a popular research direction in fire detection. This technology is widely used in outdoor fire detection fields (e.g., forest fire detection). Smoke detection is often based on features such as color, shape, texture, and motion to distinguish between smoke and non-smoke objects. However, the salience and robustness of these features are insufficiently strong, resulting in low smoke detection performance under complex environment. Deep learning technology has improved smoke detection performance to a certain degree, but extracting smoke detail features is difficult when the number of network layers is small. With no effective use of smoke motion characteristics, indicators such as false alarm rate are high in video smoke detection. To enhance the detection performance of smoke objects in videos, this paper proposes a concept of change-cumulative image by converting the YUV color space of multi-frame video images into a change-cumulative image, which can represent the motion and color-change characteristics of smoke. Then, a fusion deep network is designed, which increases the depth of the VGG16 network by arranging two convolutional layers after each of its convolutional layer. The VGG16 and Resnet50 (Deep residual network) network models are also arranged using the fusion deep network to improve feature expression ability while increasing the depth of the whole network. Doing so can help extract additional discriminating characteristics of smoke. Experimental results show that by using the change-cumulative image as the input image of the deep network model, smoke detection performance is superior to the classic RGB input image; the smoke detection performance of the fusion deep network model is better than that of the single VGG16 and Resnet50 network models; the smoke detection accuracy, false positive rate, and false alarm rate of this method are better than those of the current popular methods of video smoke detection.

## 1. Introduction

Of all disasters, fire is one of the most frequent and widespread threats to public safety and social development. Fire not only destroys material property and causes social order chaos, but also directly or indirectly endangers life. Timely fire detection helps decrease disaster risks. Therefore, studying fire detection techniques is necessary. When a fire occurs, its main form is smoke and fire. Generally, smoke occurs before fire. Thus, understanding smoke detection techniques is conducive to early fire detection and fire hazard reduction.

At present, smoke sensors are popular in indoor environments. Smoke sensors prevent fire by monitoring the smoke concentration. However, smoke sensors require smoke to enter the sensors and then detect it when the concentration reaches a certain level. Using such sensors in outdoor open spaces is difficult.

In outdoor environments, computer vision-based smoke detection technology is often used because it is not limited by space. This technology also has a large coverage area and low cost, which is one of the main directions of outdoor smoke detection research [1]. By considering the motion variation and color change of smoke and combining with the research results of deep learning technology, a video smoke detection method is proposed in this paper on the basis of change-cumulative image and fusion deep network. The main contributions are as follows:

First, the concept of change-cumulative image is put forward for the time domain transformation of YUV image. By calculating the inter-frame variation cumulative image of Y space and the mean filtering cumulative image of UV space, the change-cumulative image can represent the motion and color-change characteristics of smoke.

Second, a fusion deep network is designed. On the basis of the classic VGG16 network, the network depth is increased by multiple convolutional layer cascading. By cascading the VGG16 and Resnet50 networks, the network layers are increased, and the expression ability of the model is effectively improved, thus extracting additional identifiable characteristics of smoke.

## 2. Related Work

From a visual perspective, previous scholars often used traditional features, such as color, texture, and shape, to detect smoke. Chen et al. [2] pointed out that the gray values of smoke color in the RGB model on the three channels are close, mainly distributed within the range of 80–220. Krstinić et al. [3] proposed the HS’I model that can reflect the characteristics of smoke unlike several color models, including RGB, YCbCr, CIELab, and HSI. In addition, texture features are often used to distinguish between smoke and non-smoke objects. Gubbi et al. [4] suggested a video smoke detection method on the basis of wavelet transformation and support vector machine (SVM), which extracts a total of 60 characteristics, such as arithmetic mean, geometric mean, deviation, gradient, peak, and entropy, for describing smoke on all sub-band images of three-level wavelet decomposition. Russo et al. [5] used local binary pattern (LBP) and SVM to detect smoke in images. LBP values and histograms are calculated from the pixels of motion regions to form a feature vector for describing the texture of smoke. Yuan et al. [6] introduced a smoke detection algorithm on the basis of the multi-scale features of the LBP and LBPV pyramids. Yuan et al. [7] eliminated the shape dependency generated by the AdaBoost algorithm learning by using rules to divide the detection window, thus presenting a robust video smoke feature. However, the unfixed shape, unremarkable color, and texture of smoke often lead to extremely high false alarm rates when detecting smoke by these traditional features. Recently, deep learning technology is developing rapidly. The use of a deep network model can adaptively extract features with strong discriminating ability, which helps improve smoke detection performance [8]. Xu et al. [9] proposed a novel video smoke detection method on the basis of deep saliency network, which highlights the most important object regions in an image by using visual saliency detection method. For extracting the informative smoke saliency image, they combined the pixel- and object-level salient convolutional neural networks. Frizzi et al. [10] used CNNs (Convolutional Neural Networks) to automatically extract the characteristics of smoke with strong differentiation, which is more generalized than the characteristics of artificially selected LBP and wavelets. Zhang et al. [11] used faster R-CNN to detect smoke and produced synthetic smoke images by inserting real or simulative smoke into forest background to solve the lack of training data. Yin et al. [12] proposed a deep normalized CNN for image smoke detection (DNCNN). The network improves the convolution layer in traditional CNN to the batch standardized convolution layer, which effectively solves gradient dispersion and overfitting in the course of network training. Solving these problems speeds up the training process and improves the detection effect. In addition, the training sample data are enhanced to address the imbalance between positive and negative samples and lack of training samples. Although deep learning methods greatly improve the accuracy of smoke detection, false alarms for objects remain, such as cloud, fog, and others that are particularly similar to smoke.

For video smoke detection, the motion characteristics of smoke is the main basis of smoke detection. Toreyin et al. [13] studied the fuzzy and fluctuating characteristics of smoke over time by using wavelet transformation. Yuan et al. [14] proposed an improved fast Horn-Schunck optical flow algorithm to obtain optical flow field, by which the suspected smoke motion areas are detected. Subsequently, the smoke and other interference sources are distinguished by features of the mean direction and the average velocity of the optical flow vector. Kopilovic et al. [15] extracted the distributed entropy of the direction of motion light flow and identified the irregular characteristics of smoke movement to detect smoke. Tung et al. [16] presented a four-stage video smoke detection algorithm. In Stage 1, the motion area is extracted using an approximate median method. In Stage 2, the motion area is clustered to obtain the candidate smoke region by using the fuzzy c-means method. In Stage 3, the space-time characteristics of the candidate area are extracted. In Stage 4, the SVM is used to make a judgment. In general, the use of the motion characteristics of smoke can reduce the false alarm rate to a certain extent. However, existing methods mostly use the traditional features of smoke and motion characteristics, moreover smoke detection performance is not high enough.

## 3. Our Method

To enhance the detection accuracy of smoke objects in a video, this paper proposes a video smoke detection method on the basis of change-cumulative image and fusion deep network. First, we convert the YUV color space of multi-frame video image into a change-cumulative image, which can represent the motion and color-change characteristics of smoke. Second, we design a fusion deep network. On the one hand, the network cascades two convolutional layers after each convolutional layer of the VGG16 network to enhance feature extraction by increasing the network depth. On the other hand, the network cascades the VGG16 network with the Resnet50 network to improve the expression ability of the model while deepening the network level.

### 3.1. Change-Cumulative Image

By analyzing the smoke characteristics, the brightness of smoke varies greatly with its concentration and composition, and smoke may be bright or dark. The color of smoke slightly changes, and in general, the color saturation of smoke is very small. From the occurrence and development of smoke, it significantly diffuses upward and outward, the process of which is as follows: Smoke starts with a fire point and continues to spread up and around. On the basis of these characteristics, this paper proposes the concept of change-cumulative image, and its basic idea is as follows: The adjacent frame images in the Y space are detected for change on the YUV color space. Moreover, the variation is accumulated to obtain a change-cumulative image for reflecting the motion diffusion characteristics of smoke. The cumulative image of U and V spaces is treated with mean filtering to obtain a cumulative image for reflecting the color change of smoke. The reasons of choosing the YUV color space to build change-cumulative image are listed as below: (1) This method is mainly used to detect smoke object in videos. The color space output by camera system is usually YUV color space, which can avoid the color space transformation and reduce time consumption; (2) since the brightness of the smoke may be bright or dark, the brightness needs to be separated when selecting the color space. The Y component in the YUV color space is the luminance component, which is easy to be separated; (3) the accumulation of color components is mainly used to reduce the influence of clutter, noise, or foreground moving object which also accumulate absolute value of the difference in the Y space. In the YUV color space, the U and V components of change-cumulative image can extract deep features reflecting the color characteristics of the smoke, while other color spaces such as HSI usually has only one color channel for extracting smoke color characteristics. For example, the H component of HSI cannot be used because the chromaticity characteristics of smoke are not significant; (4) in order to facilitate the subsequent deep network construction, we hope that the change-cumulative image is similar to RGB image at the data level. For example, they all have three channels and the gray level is 255. Choosing the YUV color space to build change-cumulative image can meet this need.

In the *Y* space, the inter-frame difference method is used to calculate the binary image of the changing image. Specifically, for the kth vector image in *Y* space $Y_{k}$, the binary image of the inter-frame change image $Y_{k}^{\left(d\right)}$ can be expressed as

(1) $$Y_{k}^{\left(d\right)}(x,y)=\left\{ \begin{array}{l} \begin{array}{cc} 1 & ,\left|Y_{k}(x,y)-Y_{k-1}(x,y)\right|>T \end{array}\\ \begin{array}{cc} 0 & ,\mathrm{otherwise} \end{array} \end{array}\right.$$

where *T* is a fixed threshold. This paper takes the empirical value *T* = 10.

Then, the adjacent *N* binary images are summed to obtain a change-cumulative image $Y_{k}^{\left(a\right)}$, which is expressed as

(2) $$Y_{k}^{\left(a\right)}(x,y)=\sum_{i=k-N+1}^{k}Y_{i}^{\left(d\right)}(x,y)$$

In this paper, *N* is smaller than 255; thus, the change-cumulative image $Y_{k}^{\left(a\right)}$ can be regarded as a grayscale image. This paper takes the empirical value *N* = 100.

In the *U* space, the *U* space vector images of the adjacent *N* frame images are cumulatively summed and averaged to obtain a cumulative image $U_{k}^{\left(a\right)}$, which is expressed as

(3) $$U_{k}^{\left(a\right)}(x,y)=\frac{1}{N}\sum_{i=k-N+1}^{k}U_{i}(x,y)$$

Similarly, in the *V* space, the *V* space vector images of the adjacent *N* frame images are cumulatively summed and averaged to obtain a cumulative image $V_{k}^{\left(a\right)}$, which is expressed as

(4) $$V_{k}^{\left(a\right)}(x,y)=\frac{1}{N}\sum_{i=k-N+1}^{k}V_{i}(x,y)$$

Thus, the kth YUV image can be converted into a change-cumulative image, and its *Y* space vector can reflect the motion diffusion characteristics of smoke. The *U* and *V* space vectors reflect the color change of smoke. Compared with the original YUV image, the change-cumulative image can better highlight the changing characteristics of smoke. Therefore, on the basis of the change-cumulative image, significant and robust smoke characteristics can be extracted. For the subsequent deep network model, the cumulative image is used as the input image, in which the three-color component spaces *Y*, *U*, and *V* are the three channels of the input image that correspond to the three-color channels of the conventional RGB image.

**Figure 1:**
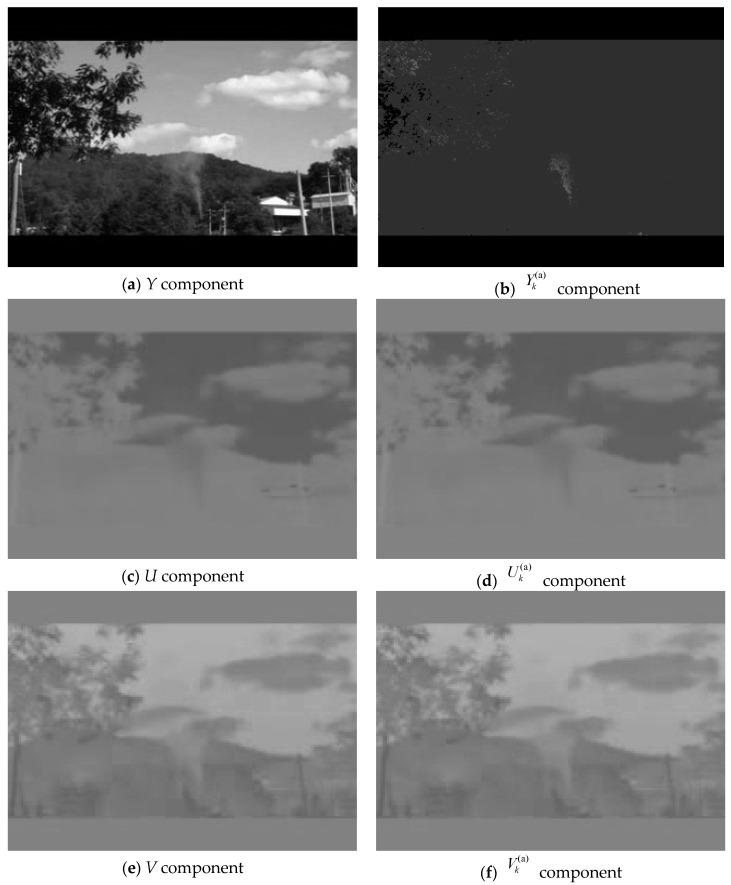
Comparison with YUV and change-cumulative image $Y_{k}^{\left(a\right)}U_{k}^{\left(a\right)}V_{k}^{\left(a\right)}$ of the 108th frame in the video “wildfire_smoke_4.avi”.

Figure 1 shows three component images of YUV and corresponding change-cumulative image $Y_{k}^{\left(a\right)}U_{k}^{\left(a\right)}V_{k}^{\left(a\right)}$ of the 108th frame in the video “wildfire_smoke_4.avi” [17]. From the *Y* component, the difference between the smoke object and the background is not obvious. However, in the $Y_{k}^{\left(a\right)}$ component of the change-cumulative image, the stationary background and the moving smoke show significant differences. From the analysis of the two components of *U* and *V*, a clear difference is found between smoke object and background. After the cumulatively summed and averaged operations, the color difference at different positions of the smoke is stable in $U_{k}^{\left(a\right)}$ and $V_{k}^{\left(a\right)}$ components. In this case, the motion and color change characteristics of the smoke in the change-cumulative image $Y_{k}^{\left(a\right)}U_{k}^{\left(a\right)}V_{k}^{\left(a\right)}$ are concentrated, which enhances the salient and robust characteristics of smoke.

### 3.2. Fusion Deep Network 

Deep networks have exhibited good performances in image classification and object detection. VGG16 is a commonly used network, which has a simple structure and can easily form a deep network. In this network structure, the convolutional and pooled layers use the same kernel function, and both layers are stacked to form a convolutional block structure for easily forming a deep network. The maximum pooling method is used between the blocks of the VGG16 network. Thus, certain important features of the original smoke images with rich details may be lost. To compensate for the lost features, this paper improves the VGG16 network by introducing a cascading convolutional layer, which enhances the extraction ability of detailed features. We also cascade the ResNet50 network and the improved VGG network for extracting more detailed features. The ResNet50 network uses a jump connection to form residual blocks, thereby conveying the image information to the deep layers of the neural network to avoid the loss of important features of smoke images. Meanwhile, this approach can avoid the under-fitting problem caused by the disappearance of the gradient and effectively improve the expression ability of the model while deepening the network level. Compared with single deep network model such as VGG16 and ResBet50, the fusion deep network proposed in this paper can extract richer detailed features of smoke images and enhance the distinguishing ability of features between smoke and non-smoke images.

(1) Cascading Convolutional Layer

Although the VGG16 network increases the extraction ability of detailed features by the combination and stacking of 3 × 3 filters, the distinguishing ability of features between smoke and smoke-like images such as cloud and fog is insufficiently strong. To further strengthen the significance of the features, this paper draws on the convolutional layer enhancement idea in literature [18], for cascading two convolutional layers after each convolutional layer of the VGG16 network.

**Figure 2:**
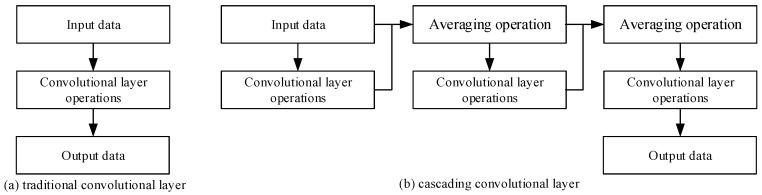
Comparison with traditional convolutional layer and cascading convolutional layer.

As illustrated in Figure 2a, after the traditional convolutional layer operations (including convolution operation, batch normalization, and reluctant activation) on the input data, the output data have the same feature dimensions as the input data.

As shown in Figure 2b, the output data after the convolution layer operation are averaged with the input data. Then, the output data after the averaging operation are used as the input data of the cascading convolutional layer to conduct a convolutional layer operation again. By analogy, three convolutional layers are cascaded, which can avoid the loss of the original detailed features during the convolution process. Such cascading also has a beneficial effect on distinguishing between smoke and smoke-like images such as cloud and fog. Concurrently, the network depth is increased by cascading, which enhances the extraction of object features and improves recognition performance. Furthermore, additional parameters need not be calculated to increase network depth because of weight sharing. Skipping the calculation can avoid the difficulties of deep training, such as over-fitting problems.

(2) Fusion Deep Network 

**Figure 3:**
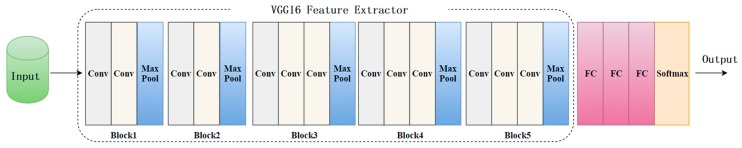
VGG16 network structure.

The VGG16 network consists of 13 convolutional layers and three fully connected layers. The network structure is displayed in Figure 3. The most notable point is that the features are extracted by the combination and stacking of 3 × 3 filters, and the distinguishing ability of features is strong.

**Figure 4:**
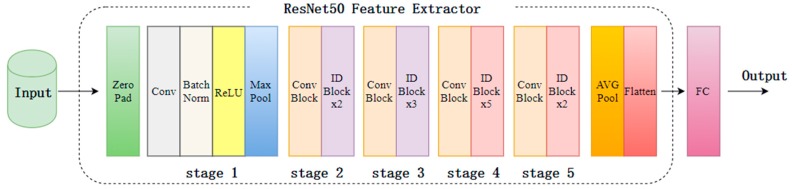
ResNet50 network structure.

The ResNet50 network consists of 49 convolutional layers and one fully connected layer. The network structure is shown in Figure 4. Given that the network joins the constant mapping layer, it directly connects the shallow network with the deep network, thereby ensuring that the network does not degrade as depth increases, and the convergence effect is good.

**Figure 5:**
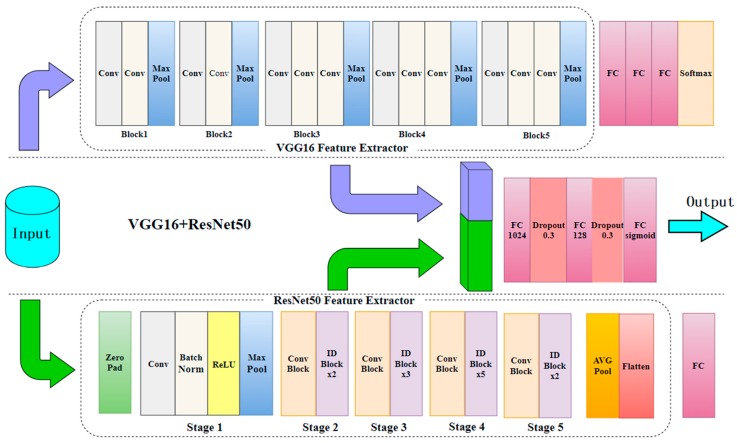
Fusion deep network structure.

In this paper, the VGG16 and ResNet50 networks are cascaded to form the fusion deep network, as illustrated in Figure 5. The details of this network structure are shown in Table 1. The VGG16 network feature extractor is the five blocks of convolutional structure, shown in the dashed box of Figure 3. The convolutional layers use the cascading convolution layers described in this paper. The ResNet50 network feature extractor is the residual block structure portion of the dashed box in Figure 4. The fusion deep network uses the VGG16 and ResNet50 feature extractors to extract features. For the input image with the size of 224 × 224, the features extracted by the VGG16 and ResNet50 feature extractors are denoted as F1 and F2, respectively. The feature dimension of F1 is 7 × 7 × 512 = 25,088, and that of F2 is 7 × 7 × 2048 = 100,352. We put F2 after F1 to construct a mixed feature denoted as F3. The feature dimension of F3 is 100,352 + 25,088 = 125,440. This feature passes through three fully connected layers and two dropout layers, and then outputs the results. The specific implementation process is as follows. Each dimension feature of the mixed feature F3 is regarded as a node of a neuron, and the extracted feature is connected by a full connection (FC), outputting 1024 neuron nodes. To prevent the over-fitting of a convolutional neural network, the dropout method is used to randomly select the neural units with a certain probability P (P = 0.3 in this paper) and discard them. Other neural units remain connected by FC to output 128 neuron nodes. The dropout operation is then performed again. Finally, the remaining neural units are output through the Sigmoid activation function. If the output value is greater than or equal to 0.5, it is determined to be a smoke object; otherwise, then it is determined to be a non-smoke object. The loss function used in this paper can be denoted as follows.

(5) $$L=xt-\max\left(x,0\right)-\ln\left(1+e^{-\left|x\right|}\right)$$

where, *t* is the label value, *x* is the input data.

The fusion deep network not only can extract the detailed features of smoke images by using the small filters of VGG16 but also can solve the feature loss and under-fitting problem of VGG16 by employing the ResNet50 network for extracting deeper features.

In summary, the Algorithm 1 of this paper is listed as follows.

**Algorithm 1.** Procedure to detect the smoke object in a video.**Input:** A video. **Initialize:**
*k* = 1, *N =* 100. **While** obtaining a frame from the input video **do**
1. using bilinear interpolation method to scale the current frame image size to 224 × 224, denoted as $Y_{k}^{}U_{k}^{}V_{k}^{}$. 2. if (*k* > = *N*) then, 3. calculating the change-cumulative image of the current frame image according to formulas (1) to (4), denoted as $Y_{k}^{\left(a\right)}U_{k}^{\left(a\right)}V_{k}^{\left(a\right)}$; 4. calculating the classification score *s* of the cumulative image after fusion deep network; 5. If (*s* > 0.5), then 6. outputting the alarm signal of smoke object; 7. End if 8. End if 9. caching images $Y_{k}^{}U_{k}^{}V_{k}^{}$ and $Y_{k}^{\left(a\right)}U_{k}^{\left(a\right)}V_{k}^{\left(a\right)}$; 10. *k* = *k* + 1; **end while**

## 4. Experiments and Results

### 4.1. Experiment Description

(1) Experimental Dataset

We test the performance of the algorithms on two public datasets of smoke video. One is the dataset provided by Prof. Yuan Feinu, which includes three smoke and three non-smoke videos [19]; the other is CVPR Lab’s video dataset, which includes four wild smoke videos and 10 non-smoke videos [17]. In our experiments, we select two smoke videos and four non-smoke videos from two datasets to build training dataset, and use the other videos to build testing dataset, the details are shown in Table 1. The total frame count of videos in training dataset is 5984, which has 3526 smoke frames. The one in testing dataset is 46,568, which has 17,084 smoke frames. All subsequent experimental results are included in the dataset described in Table 2.

(2) Evaluation Metrics

Accuracy rate (AR), true positive rate (TPR), false positive rate (FPR), and false alarm rate (FAR) are commonly used metrics for quantitatively comparing different smoke detection algorithms, denoted as follows:

(6) $$\mathrm{AR}=\frac{\mathrm{TT}+\mathrm{FF}}{T+F}$$

(7) $$\mathrm{TPR}=\frac{\mathrm{TT}}{\mathrm{TT}+\mathrm{TF}}$$

(8) $$\mathrm{FPR}=\frac{\mathrm{FT}}{\mathrm{FT}+\mathrm{FF}}$$

(9) $$\mathrm{FAR}=\frac{\mathrm{TF}}{\mathrm{TT}+\mathrm{TF}}=1-\mathrm{TPR}$$

where, TT and FT represent the number of samples with categories “True predicted to be True” and “True predicted to be False”, respectively. FF and TF represent the number of samples with categories “False predicted to be False” and ”False predicted to be True”, respectively. T = TT + FT. F = FF + TF. The category of smoke images is True, whereas that of non-smoke images is False.

(3) Experimental Environment

The experimental environment of this paper is Windows 10 system, Python 3.6.2, Tensorflow 1.11.0, and Keras 2.2.4. In the hardware device section, the graphics card is NVIDIA GeForce GTX1080Ti, and the CPU is Intel Core i7-8700K. The emulator is written in Python and uses the neural network library Keras and GPU.

### 4.2. Model Training

**Figure 6:**
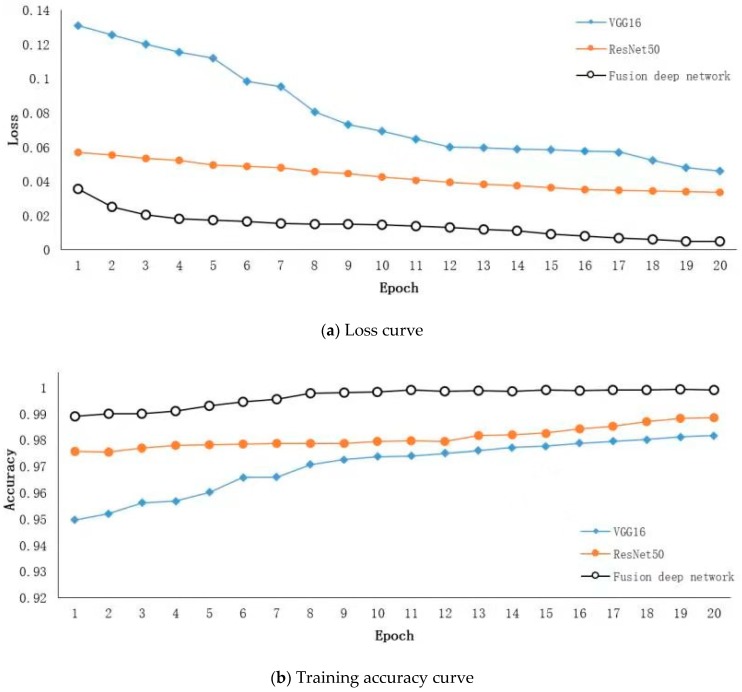
Training curves of different network models.

The following explains the training method of fusion deep network. First, we extract the change-cumulative images from the video in the training dataset. Second, we manually select the change-cumulative images of smoke and non-smoke objects to construct the positive and negative sample datasets for training the fusion deep network model, respectively. Comparatively, the number of change-cumulative images is limited. Thus, training the fusion deep network model with improved performance by relying on these images alone is difficult. This paper adopts the transfer learning method to solve the problem of small sample training, specifically by using the feature-based transfer learning method in isomorphic space. At present, few dataset and few public network models are used in smoke detection. Considering the increase in network levels, the high-level network feature layer has universality in shape and texture. Therefore, this paper uses a large amount of annotation data in the object detection domain for transfer learning. Specifically, VGG16 and ResNet50 models in the Keras database are used, and the trained pre-training weight [20] from the ImageNet dataset is used for transfer learning. Figure 6 illustrates the loss and accuracy curves in the process of training three models including fusion deep network, VGG16 network, and ResNet50 network. We use Adam (adaptive moment estimation) for optimization. The training-relevant hyper-parameters are shown in Table 3. As displayed in Figure 6a, the loss curve suggests the gap between different models under the conditions of migration-based learning and actual data, in which the loss curve of the fusion deep network model proposed in this paper can be stabilized quickly. Figure 6b shows that the training accuracy of the fusion deep network model is superior to that of the single network. The accuracy also tends to be stable at the 20th iteration. The performance of the fusion deep network proposed in this paper is better than that of the VGG16 and ResNet50 single models.

### 4.3. Performance Comparison

(1) Performance Comparison with Different Input Images and Network Models

**Figure 7:**
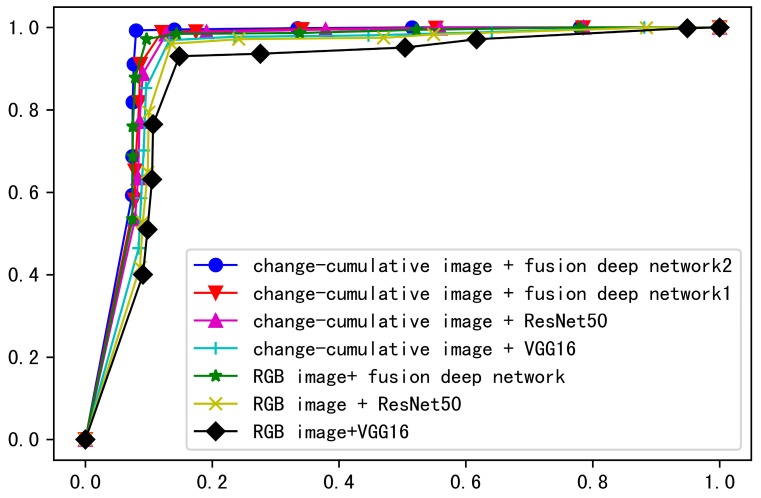
ROC curves with different input images and network models.

The main innovations of this paper include (1) the change-cumulative image, which is used as the input of the deep network model, and (2) the fusion deep network model. To verify the beneficial effects of these two innovations on smoke detection, we compare the smoke detection performance with different input images and network models. Figure 7 shows ROC (receiver operating characteristic) curves of the methods with different input images and network models. Where, “fusion deep network 1” means “fusion deep network without pre-trained ImageNet weights”, and “fusion deep network 2” means “fusion deep network with pre-trained ImageNet weights”. It is clearly that, by using change-cumulative image and fusion deep network with pre-trained ImageNet weights, we can obtain the largest AUC (area under curve) value.

**Figure 8:**
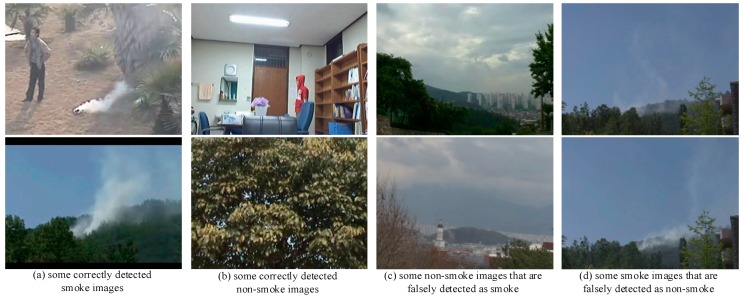
Some detection results.

The detailed results are presented in Table 4, where the classification threshold is set to 0.5. Under the same network model, the three indicators AR, FPR, and FAR of smoke detection are superior when using the change-cumulative image as the input of the network model, note that the FAR has significantly dropped. When the input image is the same, the smoke detection indicators using a fusion deep network are significantly better than those when using VGG16 or ResNet50 alone. Comparison with the fusion deep network without pre-trained ImageNet weights, the one with pre-trained ImageNet weights obtains higher AR value, at the same time gets the lower FPR and FAR values. Some detection results are shown in Figure 8. Most smoke and non-smoke images can be detected correctly, but there are still few smoke-like and smoke-insignificant objects with classification error. In general, using the change-cumulative image and the fusion deep network pre-trained ImageNet weights can greatly improve the AR value and decrease the FPR and FAR value for smoke detection, thereby enhancing the overall smoke detection performance.

(2) Performance Comparison with Different Methods

We compare the smoke detection performance of our method with that of other popular smoke detection methods in recent years, as presented in Table 5. Among them, the training and testing dataset used by all methods are the same, as shown in Table 2. The training methods and parameters of the classifier are all from the corresponding literature. Evidently, the AR value of our method is the largest, and the FPR and FAR values are the smallest. Specifically, these methods using deep learning (including CNN, DNCNN, and our method) always obtain higher AR value and lower FPR value, because the identification ability of deep features is stronger than traditional features such as color, texture, and motion. From the FAR indicator, the smoke detection methods combined with motion characteristics (including our method and literature [14]) often have a low false alarm rate, which is also caused by the unremarkable color, shape, and texture of smoke. Our method not only combines the motion, color, shape, and texture features of smoke in the change-cumulative image, but also improves the depth of network and the extraction ability of detail features by the fusion deep network. Furthermore, the smoke detection method solves the problem of insufficient training of the network model caused by small sample learning by using the transfer learning method, solving the problem improves the AR of smoke detection and decreases the FPR and FAR. However, the dfps (detected frames per second) of our method is lowest among these methods. This shows that the computational efficiency of this method is low and needs to be improved.

## 5. Conclusions

The use of computer vision technology to detect smoke in videos is a popular research direction in fire detection in recent years. Given the uncertainty of smoke objects, the traditional smoke detection methods used are impractical because of low performance, especially of the high false alarm rate. Deep learning-based methods have improved smoke detection performance to a certain degree. However, solely relying on depth characteristics results in difficulties in effectively distinguishing other objects similar to smoke, such as clouds and fog. This paper proposes a new conception of change-cumulative image, which converts the YUV color space of multi-frame video images into change-cumulative image. The change-cumulative image can describe the motion characteristics and color change of smoke. Experiments show that the change-cumulative image can improve smoke characteristics and smoke detection performance. Furthermore, a fusion deep network is designed in this paper. The main characteristics are to improve the convolutional layer of the VGG16 network and cascade two network models. Doing so can effectively improve the model expression while deepening the network level and help extract additional identifiable smoke features. The experimental results reveal that the new method can improve AR of smoke detection, especially reduce FPR and FAR, which is of great significance for the practical application of smoke detection. However, the method used in this paper extracts smoke characteristics by using two network models with complex calculation processes. The computational efficiency is low. Improving the efficiency of the algorithm is one future research direction.

## Figures and Tables

**Table 1:** The details of fusion deep network structure.

Input Layer (None, 224, 224, 3)					
VGG FEATURE Extractor			ResNet50 Feature Extractor		
Block	Layer (type)	Output Shape	Stage	Layer (type)	Output Shape
Block 1	Conv2D * 2	(None, 224, 224, 64)	Stage 1	ZeroPadding	(None, 230, 230, 3)
				Conv2D	(None, 112, 112, 64)
	MaxPooling	(None, 112, 112, 64)		BatchNormalization	(None, 112, 112, 64)
				MaxPooling	(None, 56, 56, 64)
Block 2	Conv2D * 2	(None, 112, 112, 128)	Stage 2	$\left[\begin{array}{cc} 1*1 & 64\\ 3*3 & 64\\ 1*1 & 256 \end{array}\right]*3$	(None, 56, 56, 256)
	MaxPooling	(None, 56, 56, 128)			
Block 3	Conv2D * 3	(None, 56, 56, 256)	Stage 3	$\left[\begin{array}{cc} 1*1 & 128\\ 3*3 & 128\\ 1*1 & 512 \end{array}\right]*4$	(None, 28, 28, 512)
	MaxPooling	(None, 28, 28, 256)			
Block 4	Conv2D * 3	(None, 28, 28, 512)	Stage 4	$\left[\begin{array}{cc} 1*1 & 256\\ 3*3 & 256\\ 1*1 & 1024 \end{array}\right]*6$	(None, 14, 14, 1024)
	MaxPooling	(None, 14, 14, 512)			
Block 5	Conv2D * 3	(None, 14, 14, 512)	Stage 5	$\left[\begin{array}{cc} 1*1 & 512\\ 3*3 & 512\\ 1*1 & 2048 \end{array}\right]*3$	(None, 7, 7, 2048)
	MaxPooling	(None, 7, 7, 512)			
Concatenate (None, 7, 7, 2560)					
Flatten (None, 125400)					
Fc & dropout 0.3 (None, 1024)					
Fc & dropout (None, 128)					
Output Fc & sigmoid (None, 1)					

**Table 2:** Description of the dataset used in this paper.

Dataset	Description	Names
training dataset	smoke videos	Dry_leaf_smoke_02.avi [19] wildfire_smoke_1.avi [17]
	non-smoke videos	Waving_ leaves_895.avi [19] smoke_or_flame_like_object_1.avi, smoke_or_flame_like_object_2.avi, smoke_or_flame_like_object_3.avi [17]
testing dataset	smoke videos	Cotton_rope_smoke_04.avi, Black_smoke_517.avi [19] wildfire_smoke_2.avi, wildfire_smoke_3.avi, wildfire_smoke_4.avi [17]
	non-smoke videos	Traffic_1000.avi, Basketball_yard.avi [19] smoke_or_flame_like_object_4.avi, smoke_or_flame_like_object_5.avi, smoke_or_flame_like_object_6.avi, smoke_or_flame_like_object_7.avi, smoke_or_flame_like_object_8.avi, smoke_or_flame_like_object_9.avi, smoke_or_flame_like_object_10.avi [17]

**Table 3:** Training-relevant hyper-parameters.

Hyper-Parameters	α	β_1_	β_2_	ε
Value	0.001	0.9	0.999	10e-8

**Table 4:** Comparisons with different input images and network models.

Input Image	Network	AR/%	FPR/%	FAR/%
RGB image	VGG16	88.06	14.82	6.97
RGB image	ResNet50	89.98	13.54	3.94
RGB image	fusion deep network	92.86	9.64	2.82
change-cumulative image	VGG16	90.48	13.24	3.09
change-cumulative image	ResNet50	91.33	12.74	1.64
change-cumulative image	fusion deep network (without pre-trained ImageNet weights)	91.96	12.05	1.12
change-cumulative image	fusion deep network (with pre-trained ImageNet weights)	94.67	7.99	0.73

**Table 5:** Comparisons with different methods.

Methods	AR/%	FPR/%	FAR/%	dfps
HS’I model [3]	62.68	48.58	17.88	137
LBP + SVM [5]	81.17	25.60	7.14	57
CNN [10]	88.36	16.58	3.05	4
DNCNN [11]	89.49	14.37	3.84	2
dynamic characteristics [14]	81.18	28.14	2.72	27
our method	94.67	7.99	0.73	1
